# Universal representation learning for multivariate time series using the instance-level and cluster-level supervised contrastive learning

**DOI:** 10.1007/s10618-024-01006-1

**Published:** 2024-02-09

**Authors:** Nazanin Moradinasab, Suchetha Sharma, Ronen Bar-Yoseph, Shlomit Radom-Aizik, Kenneth C. Bilchick, Dan M. Cooper, Arthur Weltman, Donald E. Brown

**Affiliations:** 1 Department of Engineering Systems and Environment, University of Virginia, Charlottesville, VA 22904, USA; 2 School of Data Science, University of Virginia, Charlottesville, VA 22904, USA; 3 Pediatric Exercise and Genomics Research Center, University of California, Irvine, CA 92697, USA; 4 Pediatric Pulmonary Institute, Ruth Rappaport Children’s Hospital, Rambam Health Care Campus, 3109601 Haifa, Israel; 5 Cardiovascular Division, Department of Medicine, University of Virginia Health System, Charlottesville, VA 22903, USA; 6 Institute for Clinical and Translational Science, University of California, Irvine, CA 92697, USA; 7 Department of Kinesiology, University of Virginia, Charlottesville, VA 22903, USA; 8 Division of Endocrinology and Metabolism, Department of Medicine, University of Virginia, Charlottesville, VA 22903, USA

**Keywords:** Multivariate time series data, Contrastive learning, Classification, Interpretability

## Abstract

The multivariate time series classification (MTSC) task aims to predict a class label for a given time series. Recently, modern deep learning-based approaches have achieved promising performance over traditional methods for MTSC tasks. The success of these approaches relies on access to the massive amount of labeled data (i.e., annotating or assigning tags to each sample that shows its corresponding category). However, obtaining a massive amount of labeled data is usually very time-consuming and expensive in many real-world applications such as medicine, because it requires domain experts’ knowledge to annotate data. Insufficient labeled data prevents these models from learning discriminative features, resulting in poor margins that reduce generalization performance. To address this challenge, we propose a novel approach: supervised contrastive learning for time series classification (SupCon-TSC). This approach improves the classification performance by learning the discriminative low-dimensional representations of multivariate time series, and its end-to-end structure allows for interpretable outcomes. It is based on supervised contrastive (SupCon) loss to learn the inherent structure of multivariate time series. First, two separate augmentation families, including strong and weak augmentation methods, are utilized to generate augmented data for the source and target networks, respectively. Second, we propose the instance-level, and cluster-level SupCon learning approaches to capture contextual information to learn the discriminative and universal representation for multivariate time series datasets. In the instance-level SupCon learning approach, for each given anchor instance that comes from the source network, the low-variance output encodings from the target network are sampled as positive and negative instances based on their labels. However, the cluster-level approach is performed between each instance and cluster centers among batches, as opposed to the instance-level approach. The cluster-level SupCon loss attempts to maximize the similarities between each instance and cluster centers among batches. We tested this novel approach on two small cardiopulmonary exercise testing (CPET) datasets and the real-world UEA Multivariate time series archive. The results of the SupCon-TSC model on CPET datasets indicate its capability to learn more discriminative features than existing approaches in situations where the size of the dataset is small. Moreover, the results on the UEA archive show that training a classifier on top of the universal representation features learned by our proposed method outperforms the state-of-the-art approaches.

## 1 Introduction

The goal of time series classification (TSC) is to predict the class label for a given time series data, which is a sequence of real-value observations ordered by time. While most state-of-the-art methods proposed for TSC have focused on univariate TSC, where each case consists of a single series (i.e., one dimension), real-world time series datasets in many applications are multivariate-containing multiple dimensions but a single label. With the advancement of sensor technologies, the Multivariate Time Series Classification (MTSC) problem has received great attention in a wide range of research domains and applications such as Human Activity Recognition (Minnen et al. 2006), EEG/ECG data analysis (Wang et al. 2015), and Motion Recognition (Rakthanmanon and Keogh 2013).

An ideal TSC method should be accurate, efficient, and interpretable. However, even accurate state-of-the-art TSC models suffer from a lack of interoperability or efficiency. Most general TSC approaches involve a preliminary learning phase to extract feature candidates from the time series data, such as a bag of patterns (Senin and Malinchik 2013) or time series shapelet (Ye and Keogh 2009). These methods become less computationally efficient when dealing with long-time series data as selecting features from a larger feature space increases the computational complexity of the model. The challenge is amplified in the multivariate case, where feature selection from a vast feature space becomes more difficult (Zhang et al. 2020). Recently, ensemble methods have achieved high accuracy for TSC tasks, while their computational complexity increases with the number of time steps and dimensions. For instance, the Hierarchical Vote Collective of Transformation-based Ensembles (HIVE-COTE) (Lines et al. 2016), has high training complexity $O\left(N^{2}\cdot T^{4}\right)$, as highlighted by (Lucas et al. 2019), where T represents the length of the series and N is the number of dimensions. The latest version, HIVE-COTE v2.0, (Middlehurst et al. 2021) for multivariate data requires a substantial run time (Ruiz et al. 2021). However, studies indicate that deep learning models significantly surpass HIVE-COTE in terms of run time. Importantly, these methods do not provide interpretable results.

Recently, deep learning-based methods with cross-entropy loss function have demonstrated promising performance in TSC tasks (e.g. ResNet (Wang et al. 2017), Inception (Ismail Fawaz et al. 2020). One of the main advantages of the deep learning approaches is their capability to manage large feature spaces by learning low-dimensional feature representations (Zhang et al. 2020). Moreover, these approaches require less domain-specific knowledge compared to the traditional methods for handling time series data. However, these advantages come at the cost of a substantial requirement for a large amount of labeled data during training, posing challenges when dealing with time series data that has limited labeling. Zhang et al. (2020) suggested that the traditional TSC models can effectively mitigate the issue of limited data by using distance-based methods. They proposed the TapNet deep learning model (Zhang et al. 2020) with a distance-based loss function instead of a cross-entropy loss function to address the issue of limited data.

To enable deep learning models to handle limited labelings in TSC tasks while learning the low-dimensional feature representations, we propose the *supervised contrastive learning* for *time series classification* (SupCon-TSC) model. It is based on supervised contrastive learning (SupCon) and provides interpretable outcomes. The recent success of the SupCon learning approach in various computer vision tasks inspired us to adapt this competitive approach for the TSC tasks. The SupCon loss function overcomes the shortcomings of the cross-entropy loss function, such as a lack of robustness to noisy labels (Zhang and Sabuncu 2018; Sukhbaatar et al. 2014) and the potential for decision boundaries with poor margins resulting in poor classification performance. Leveraging the SupCon learning approach alleviates the challenge of defining classification boundaries between classes. It achieves this by bringing the representations of instances with the same label closer together while moving them farther from those with different labels. In addition, because the SupCon loss function is a distance-based loss, it effectively addresses the issue of limited data in time series tasks. However, despite the advantages of the SupCon loss function, the intra-class variances and inter-class similarities found in many real-world time series make it challenging to learn universal low-dimensional feature representations using SupCon loss. To address this issue, we extend the SupCon learning approach by proposing to learn the low-dimensional universal representation, not only by applying the SupCon loss between time series instances but also between the clusters of instances across batches, as depicted in Fig. 2. In this approach, we cluster the time series instances based on their labels within each batch. Subsequently, we apply the SupCon learning approach between each instance and centers of generated clusters across batches. This introduces cluster-level SupCon as a complement to an instance-level contrastive strategy. We introduce a cluster memory bank that allows us to access representations of clusters generated in previous batches during training. This approach helps in bringing clusters with the same label closer and distancing those with different labels. This process results in clearer boundary decisions by reducing intra-class variances and inter-class similarities. Unlike existing contrastive loss function studies, our proposed approach does not depend on designing complex augmentation methods, which are challenging for time series data. The temporal dependencies in time series data present challenges in designing augmentation methods. This complexity is amplified when dealing with the MTSC task, as it requires considering the cross-correlations between variables across time. The major contributions of this paper are summarized as follows:

1. We proposed SupCon-TSC for time series data to capture contextual information, which provides interpretable outputs.
2. Even though the contrastive objective is usually based on augmented context views to get good results, the proposed approach does not depend on adopting well-known augmentation methods. In other words, the proposed approach is capable of learning the universal low-dimensional feature representations without introducing undetected inductive bias created by adopting well-known augmentation methods such as transformation- and cropping-invariance.
3. We evaluate the performance of the SupCon-TSC model on two small CPET datasets to demonstrate the model’s capability for learning better discriminative features than existing models.
4. We conduct extensive experiments on multivariate time series data to show the effectiveness of our method compared to standard approaches in the literature. Our new approach outperforms existing SOTAs on 29 UEA Archive datasets.
5. We design a SupCon loss at the cluster level in addition to the instance level to alleviate the negative impact induced by intra-class variances and inter-class similarities during training.

The rest of the paper is structured as follows: Section 2 presents the related work in MTSC, and our new model is introduced in Sect. 3. Section 4 discusses the experimental results on two CPET datasets and UEA Archive datasets, and the summary of the research is presented in Sect. 5.

## 2 Related works

In this section, we discuss relevant related work in the area of time-series classification. The state-of-the-art MTS classifiers are generally categorized into three groups: similarity-based, feature-based, and deep learning methods.

The similarity-based approaches typically utilize a similarity function such as Euclidean distance (Keogh and Kasetty 2003), edit distance (Chen et al. 2005), wavelets (Chan and Fu 1999), and Dynamic Time Warping (DTW) (Senin 2008) to measure the similarity between two instances. In these approaches, the new time series instance is classified best on its similarity to the top-k neighbors in the historical data. DTW is the most popular distance function, and two versions of it for MTSC are the independent (*DTW*_*I*_) and dependent approaches (*DTW*_*D*_) (Shokoohi-Yekta et al. 2017). The independent strategy defines a different point-wise distance matrix for each dimension and then sums them up. In contrast, the dependent strategy performs warping over all the given dimensions simultaneously by calculating the Euclidean distance between vectors containing all dimensions.

On the other hand, conventional feature-based classification methods involve the manual design of feature extraction algorithms combined with machine learning models for classification. Based on the literature, Shapelets-based (gRSF (Karlsson et al. 2016) and UFS (Wistuba et al. 2015)) and Bag of Word-based classifiers (LPS (Baydogan and Runger 2016), mv-ARF (Tuncel and Baydogan 2018), SMTS (Baydogan and Runger 2015) and WEASEL+MUSE (Schäfer and Leser 2017)) are two popular feature-based algorithms. To classify time series data, Shapelets-based models transform the original time series into a lower-dimensional space by using subsequences. However, Bag of Word-based classifiers perform the classification by converting time series into a Bag of Words (BoW) and building a classifier upon the BoW representation. Recently, the WEASEL+MUSE (Schäfer and Leser 2017) model, which uses the bag of Symbolic Fourier Approximation (SFA) symbol model, outperforms gRSF, LPS, mv-ARF, SMTS, and UFS. However, both shapelets-based and BoW-based methods are computationally expensive and have a long learning process (He et al. 2022).

Recently, deep learning techniques (XCM (Fauvel et al. 2021), FCN (Wang et al. 2017), MLSTM-FCN (Karim et al. 2019), MTEX-CNN (Assaf et al. 2019), ResNet (Wang et al. 2017), and TapNet (Zhang et al. 2020)) have been used extensively for time series classification. These techniques offer the advantage of automatically extracting the important features from time-series data for classification, as opposed to the feature-based methods listed above that require significant manual effort. However, a large amount of data is needed to train these models. These techniques commonly contain the stack of CNN layers and LSTM layers to extract features along with the softmax layer to predict the label. We describe these techniques briefly below. However, Ismail Fawaz et al. (2019) provides a more elaborate survey. Karim et al. (2019) proposed a model named MLSTM-FCN which consists of an LSTM layer and a stacked CNN layer to extract features.

Assaf et al. (2019) proposed MTEX-CNN, which utilizes a sequence of 2D and 1D convolution filters to extract MTS features corresponding to the observed variables and time, respectively. However, this model has some limitations which have been addressed by Fauvel et al. (2021). Fauvel et al. (2021) propose the XCM model, which uses the 2D and 1D convolution filters parallelly over the input data to extract features corresponding to observed variables and time, separately.

Even though deep learning-based methods can learn the latent features by training convolutional or recurrent networks, they require large-scale labeled data. Recently, Zhang et al. (2020) proposed the TapNet model with a distance-based loss function instead of a cross-entropy loss function to address the issue of limited data. None of the existing work addresses the problem of the limited labeled data, except TapNet.

One of the works most closely related to our proposed SupCon-TSC model is TS2Vec (Yue et al. 2022), which also leverages contrastive learning to capture robust contextual representations for arbitrary time steps and sub-series of the original time series, for a wide range of tasks including univariate and multivariate time series classification. TS2Vec employs hierarchical contrasting to discriminate between positive and negative samples at both instance-wise and temporal dimensions. This allows it to capture contextual representations at varying granularities while imposing the constraint of contextual consistency. In addition, it imposes the constraint of contextual consistency that states that representations for the same sub-series in two different augmented contexts should be consistent, ensuring robustness. The key differences between SupCon-TSC and TS2Vec lie in how we apply contrastive loss at both the instance level and the cluster level. The use of cluster-level contrastive loss is advantageous as it mitigates the negative impact caused by intra-class variances and inter-class similarities during training. Moreover, the SupCon-TSC model is based on supervised contrastive learning whereas TS2Vec is an unsupervised learning approach. The incorporation of the supervised contrastive (SupCon) loss in our model’s supervised learning setting encourages the extraction of more distinguishable features between different classes. This is because the loss function is designed to learn the similarity function. Additionally, the SupCon-TSC model effectively addresses the challenge of limited data in time series tasks due to its distance-based loss nature.

## 3 Methodology

In this section, we first provide a brief introduction to the problem formulation in Sect. 3.1. Following that, we elaborate on the details of the proposed method and our framework in Sect. 3.2.

### 3.1 Problem formulation

In multivariate time series classification, a data set consists of pairs (𝒳,y), where $𝒳=\left\{X_{1},X_{2},X_{3},\ldots ,X_{n}\right\}\in R^{n\times m\times l}$ contains n multi-dimensional time series observations and $y\in R^{n}$ contains corresponding discrete class variables with c possible values for each observation. Here, each time series observation can be represented as a matrix with the dimension m and time series length l. The goal of the MTSC tasks is to train a classifier on the observed pairs of (𝒳,y), enabling it to predict the class label of a new, unlabeled time series observation.

### 3.2 New model

**Fig. 1:**
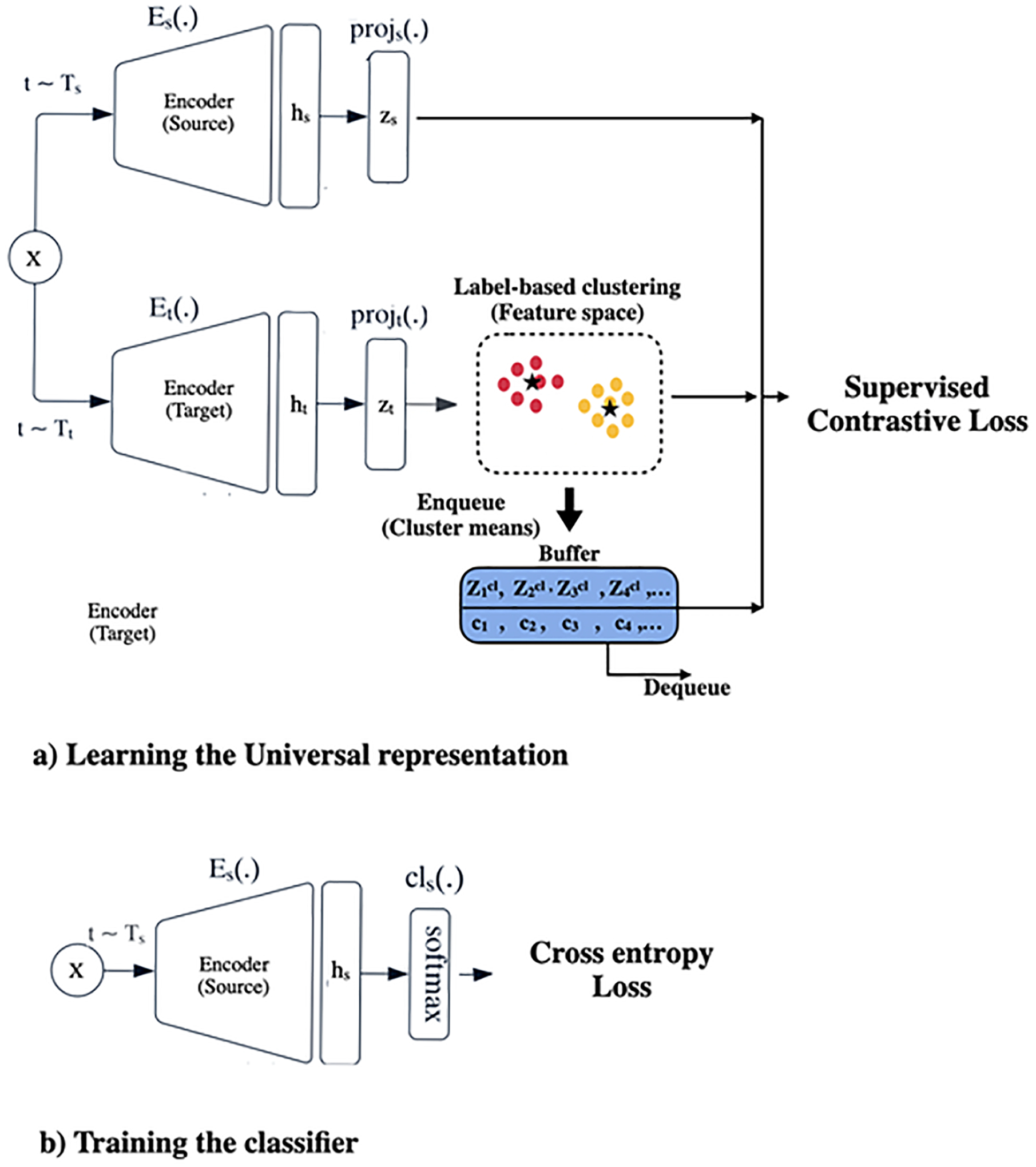
Diagram of training process

In this section, we introduce our novel approach, i.e., SupCon-TSC, which aims to enhance model performance for downstream tasks like classification by learning a universal representation for multivariate time series data. The proposed approach consists of two stages: a) Learning the universal representation, and b) Training the classifier, as depicted in Fig. 1. The first stage of SupCon-TSC is built upon the SupCon framework (Khosla et al. 2020), initially designed for image representation learning. However, we have made modifications to adapt it to learning a universal representation of multivariate time series data for supervised MTSC. Algorithm 1 outlines the pseudo-code for this first stage. Specifically, the provided pseudo-code outlines an algorithm for learning a universal representation for multivariate time series data using instance-level and cluster-level supervised contrastive learning. The algorithm begins by initializing hyperparameters, encoder, and projection head weights, and creating an empty buffer. During the training process, as the algorithm progresses through a fixed number of epochs $\left(N_{e}\right)$, a check is performed to determine whether the current epoch falls within the warm-up period $(\left.N_{w}\right)$ (i.e., lines 3 to 7). If the current epoch is within the warm-up period, the variable α is set to 0, implying that the cluster-level contrastive learning step is skipped. However, if the current epoch is equal to or greater than the number of warm-up epochs, α is set to 1, indicating that the cluster-level contrastive learning step will be executed as part of the algorithm for that epoch. The algorithm then iterates over sampled mini-batches, as depicted in lines 2–37. For each instance in the mini-batch, the algorithm applies augmentation techniques to generate weak $\left(x_{k}^{w}\right)$ and strong $\left(x_{k}^{s}\right.)$ views of the given input sequence (i.e., lines 10 and 11). Lines 12–15 demonstrate that the encoder processes these augmented sequences, and the projection head projects their hidden representations into lower-dimensional feature vectors. The algorithm performs clustering on the instances in the mini-batch based on their labels according to lines 16–18. Each instance is assigned to the cluster with the same label. As observed in lines 20–23, for each unique label, the algorithm calculates the average feature vector of instances $\left(z_{i}^{cl}\right)$ with the associated label $\left(c_{k}\right)$ and adds it to the buffer along with the corresponding label. The algorithm then proceeds to compute the instance-level and cluster-level contrastive losses in lines 25–36. More details on Learning the Universal Representation, instance-level, and cluster-level contrastive learning approaches have been provided in the following sections.

The second stage of SupCon-TSC contains training the multilayer perceptron (MLP) classifier on top of the frozen representations using a cross-entropy loss.

**Algorithm 1:** Proposed instance-level and cluster-level SupCon algorithm

**Input**: Input multi-dimension time series instances (X), Labels (Y)	
**Parameter**: Buffer size (β), Batch size (N), Number of epochs $\left(N_{e}\right)$, Number of warm-up epochs ($N_{w}$) Number of unique labels $\left(N_{l}\right)$, Temperature (τ),	
1:	Initialize the weights of the encoder (f) and projection head (g), Initialize buffer (B).
2:	**for** $\text{epoch}:1:N_{e}$ **do**
3:	**if** $\text{epoch}<N_{w}$ **then**
4:	α=0.
5:	**else**
6:	α=1.
7:	**end if**
8:	**for** sampled mini-batch **do**
9:	**for** k∈1,…,N **do**
10:	$x_{k}^{s}=T_{s}\left(x_{k}\right)$
11:	$x_{k}^{t}=T_{t}\left(x_{k}\right)$
12:	$h_{k}^{s}=E\left(x_{k}^{s}\right)$
13:	$h_{k}^{t}=E\left(x_{k}^{t}\right)$
14:	$z_{k}^{s}=\text{proj}\left(h_{k}^{s}\right)$
15:	$z_{k}^{t}=\text{proj}\left(h_{k}^{t}\right)$
16:	**Cluster instances in the batch**
17:	Assign each time series instance ($x_{k}$) to the cluster with the
18:	same label ($c_{k}$) od
19:	**end for**
20:	**for $i\in 1,\ldots ,N_{l}$ do**
21:	$z_{i}^{cl}=\frac{\sum_{k=1}^{N}I\left\{c_{k}=i\right\}z_{k}^{t}}{\sum_{k=1}^{N}I\left\{c_{k}=i\right\}}$
22:	Update the Buffer B by adding $z_{i}^{cl}$ and corresponding lable $c_{k}$
23:	to it
24:	**end for**
25:	**for** k∈1,…,N **do**
26:	**Instance-level SupCon**
27:	A(k)={1,…,N}
28:	$P(k)=\left\{p\in A(k):y_{k}=y_{p}\right\}$
29:	$L_{k}^{Ins-\text{level}}=\frac{-1}{\text{P}(\text{k})}\sum_{p\in P(k)}log\frac{exp\left(z_{k}^{s}\cdot z_{p}^{t}/\tau \right)}{\sum_{a\in A(i)}\text{exp}\left(z_{k}^{S}\cdot z_{a}^{t}/\tau \right)}$
30:	**Cluster-level SupCon**
31:	$A_{buf}(k)=\{1,\ldots ,\beta \}$
32:	$P_{buf}(k)=\left\{p\in A_{buf}(k):y_{k}=y_{p}\right\}$
33:	$L_{k}^{\text{clus–level}}=\frac{-1}{\left|P_{buf}(k)\right|}\sum_{p\in P_{buf}(k)}log\frac{exp\left(z_{k}^{s}\cdot z_{p}^{\text{clus}}/\tau \right)}{\sum_{a\in A_{buf}(i)}exp\left(z_{k}^{S}\cdot z_{a}^{\text{clus}}/\tau \right)}$
34:	**end for**
35:	$L=\sum_{k=1}^{N}L_{k}^{\text{Ins–level}}+\alpha L_{k}^{\text{cl–level}}$
36:	**end for**
37:	**end for**

#### 3.2.1 Learning the universal representation

This stage serves as the pre-training phase for training the encoder to generate the universal representation. As depicted in Fig. 1a, the Siamese network consists of source $\left(E_{s}\right)$ and target encoders $\left(E_{t}\right)$, which take two augmented versions of a multivariate time series instance sampled from two distinct augmentation families.

$$x^{s}\sim T_{s}(x)$$

$$x^{t}\sim T_{t}(x)$$

where, $x^{s}$, and $x^{t}$ represent the strongly and weakly augmented view of x, respectively. The high-variance strong augmentation $\left(T_{s}\right)$ and low-variance weak augmentation $\left(T_{t}\right)$ families are used to generate these strongly and weakly augmented views of x for the source and target networks, respectively. Wang et al. (2022) demonstrated that these settings enhance the model performance on downstream tasks such as classification. Noted, even though an essential part of the success of the contrastive learning methods is designing and utilizing good data augmentation methods (Grill et al. 2020), our approach does not depend on the well-known augmentation methods. We propose to use only jittering augmentation with low variance (weak augmentation) for the target network and high variance (strong augmentation) for the source network. After generating the augmented views of a given instance (x), they are passed to the encoder to learn the universal low dimensional representations (h=E(x)). To train the encoder, first, the encoder output will be sent to the MLP projection head to obtain the normalized embedding (z = proj(E(x)). In each iteration, the buffer is updated with the output from the target network. For every iteration, the target outputs of the given batch are clustered according to their labels, and the buffer is updated with the mean value of the clusters. Subsequently, the SupCon loss is calculated between the output of the source network, the output of the target network, and the buffer. This process aims to learn a discriminative representation that effectively characterizes instance x. The SupCon loss function enforces the normalized embeddings from the same class to pull closer together than embeddings from different classes. For this purpose, it tries to maximize the dot product between the given anchor and positive samples (i.e., samples with the same labels) while minimizing the dot product with negative samples (i.e., samples with different labels) within the batch. The SupCon learning is conducted at the instance and cluster level, which are explained in the following sections in detail.

#### 3.2.2 Supervised contrastive learning at the instance-level

As depicted in Algorithm 1, within a batch of N samples, two encoding representations are generated for each instance: the source encoding representation $\left(z^{s}\right)$ and the target encoding representation $\left(z^{t}\right)$. We expect the source encoding to have higher variance in comparison with the target encoding representation as we use higher variance in the corresponding augmentation method.

The instance-level Supervised contrastive loss is as follows:

(1) $$L^{\text{SupCon}}=\frac{-1}{\left|P(k)\right|}\sum_{p\in P(k)}log\frac{exp\left(z_{k}^{s}\cdot z_{p}^{t}/\tau \right)}{\sum_{a\in A(i)}exp\left(z_{k}^{s}\cdot z_{a}^{t}/\tau \right)}$$

where, τ is the temperature. For an anchor embedding $z_{k}^{s}$ that comes from the source network, we denote $z_{p}^{t}$ as a positive sample which is the output of the target network corresponding to the sample in the batch with the same label as the anchor image. Hence, $\left(z_{k}^{s},z_{p}^{t}\right)$ is a positive pair and the number of positive pairs for the anchor k is equal to the number of instances with the same label as the anchor instance in the batch. A(i) is a set of all indexes in the given batch, while P(k) indicates a set of positive samples for the anchor k. *P*(*k*) contains indexes of those samples in the batch which have the same label as the anchor k.

**Fig. 2:**
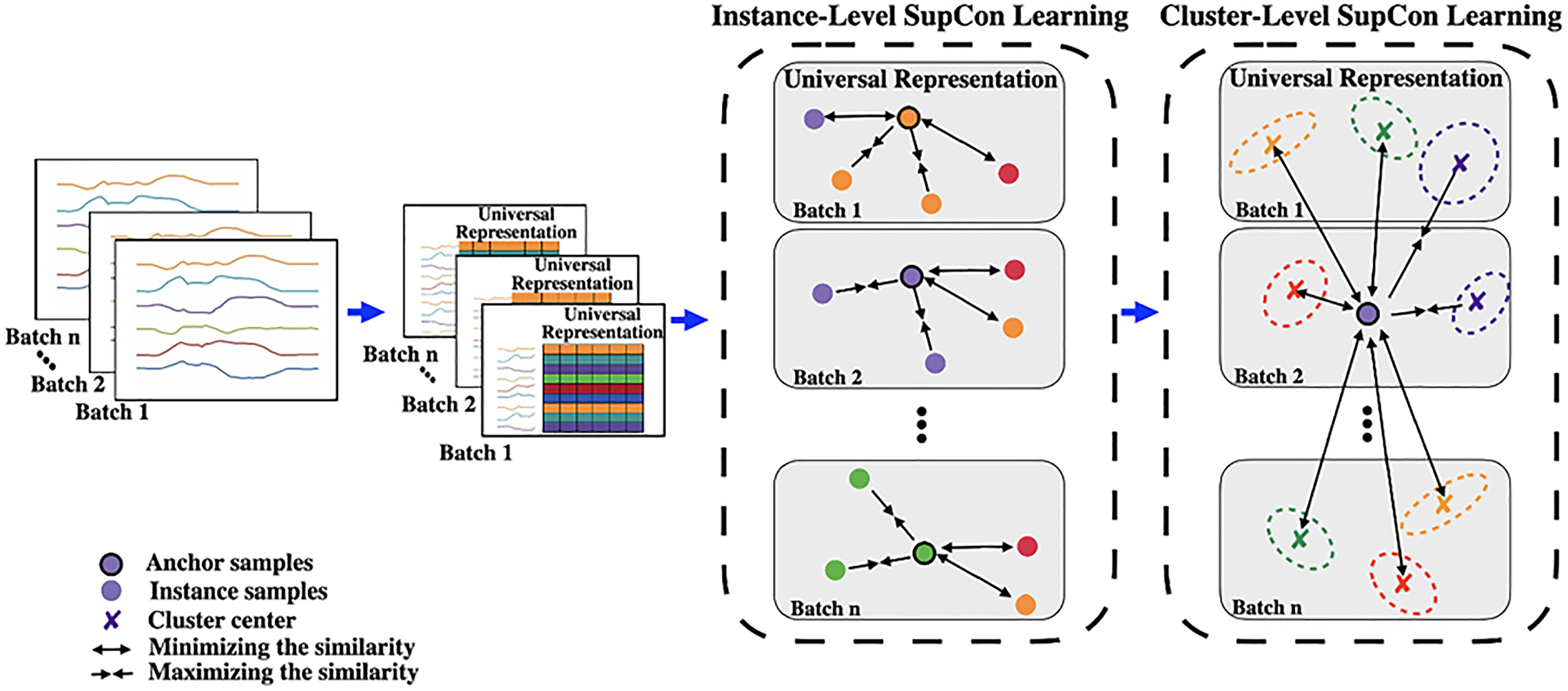
Diagram of proposed approach

Noted, the size of negative samples for the anchor k is N(k)=|A(i)|-|P(k)|. Figure 2 presents the Instance-level supervised contrastive learning between a given anchor and positive and negative samples in each batch.

#### 3.2.3 Supervised contrastive learning at the cluster-level among batches

In this approach, we propose a cluster memory bank that contains the representation of the cluster’s center generated in the previous batches during training. In each batch with N samples, we perform clustering over the target embeddings based on their labels. We assign the target embedding of each time series sample $x_{k}$ to the cluster with the same label $\left(c_{k}\right)$. Then, we determine the cluster centers using Eq. (2). The representations of the cluster centers generated in each batch will be stored in the cluster memory bank. The cluster memory bank is built with size $N_{\text{buffer}}\times N_{l}\times D$, where $N_{\text{buffer}},N_{l}$, and D are the memory size, number of unique classes for time series data set and the dimension of representation embedding, respectively.


(2) $$z_{i}^{cl}=\frac{\sum_{k=1}^{N}I\left\{c_{k}=i\right\}z_{k}^{t}}{\sum_{k=1}^{N}I\left\{c_{k}=i\right\}}$$

As shown in Algorithm 1, the cluster-level SupCon learning is conducted using Eq. (3) among the batches during training in addition to the instance-level SupCon learning in each batch.


(3) $$L_{k}^{\text{clus–level}}=\frac{-1}{\left|P_{buf}(k)\right|}\sum_{p\in P_{buf}(k)}log\frac{exp\left(z_{k}^{s}\cdot z_{p}^{\text{clus}}/\tau \right)}{\sum_{a\in A_{buf}(i)}\text{exp}\left(z_{k}^{s}\cdot z_{a}^{\text{clus}}/\tau \right)}$$

We aim to optimize the following objectives: 1) Maximize the similarity between each instance embedding in a batch $z_{k}^{s}$ and positive samples $z_{p}^{\text{clus}}$ retrieved from the cluster memory bank, 2) Minimizing the similarity between each instance embedding in a batch $z_{k}^{s}$ and negative samples also sourced from the cluster memory bank. In Eq. (3), $A_{\text{buf}}(i)$ denotes the set of all indexes within the cluster memory bank, while $p_{buf}(k)$ represents the set of positive samples which have the same label as the anchor k in the cluster memory bank. Figure 2 outlines the cluster-level SupCon learning approach, depicting the interaction between a given anchor instance and positive and negative samples (i.e. centers of the clusters with the same and different labels) extracted from the cluster memory bank. The overall piece-wise training loss can be defined as follows:

(4) $$L=\sum_{k=1}^{N}L_{k}^{\text{Ins–level}}+\alpha L_{k}^{\text{cl–level}}$$

(5) $$\alpha =\left\{\begin{array}{l} 0\;\text{epoch}\;\leq N_{w}\\ 1\;\text{epoch}\;>N_{w} \end{array}\right.$$

We only utilize the instance-level contrastive loss to train the model during the first epochs. After training the model for $N_{w}$ epochs, we take into account the cluster-level loss in addition to the instance-level loss to train the model.

#### 3.2.4 Training the classifier

Illustrated in Fig. 1b, the objective of the second stage is to train a classifier on top of the source encoder, utilizing cross-entropy loss for predicting class labels in MTSC tasks. During this step, we discard the projection head (Proj(.)), and the classifier is incorporated into the preserved frozen universal representation. Subsequently, the classifier is trained using the cross-entropy loss function.

## 4 Experiments

In this section, we assess the performance of SupCon-TSC on three different datasets: the UEA multivariate time series archive dataset and two cardiopulmonary exercise testing datasets. Firstly, we provide detailed descriptions of the datasets, metrics used for evaluation, and the implementation specifics. Subsequently, we present a comprehensive analysis of experimental results, comparing the performance across diverse datasets. Finally, we delve into the ablation studies section, conducting in-depth analyses to further understand the model’s effectiveness.

### 4.1 Datasets

**Fig. 3:**
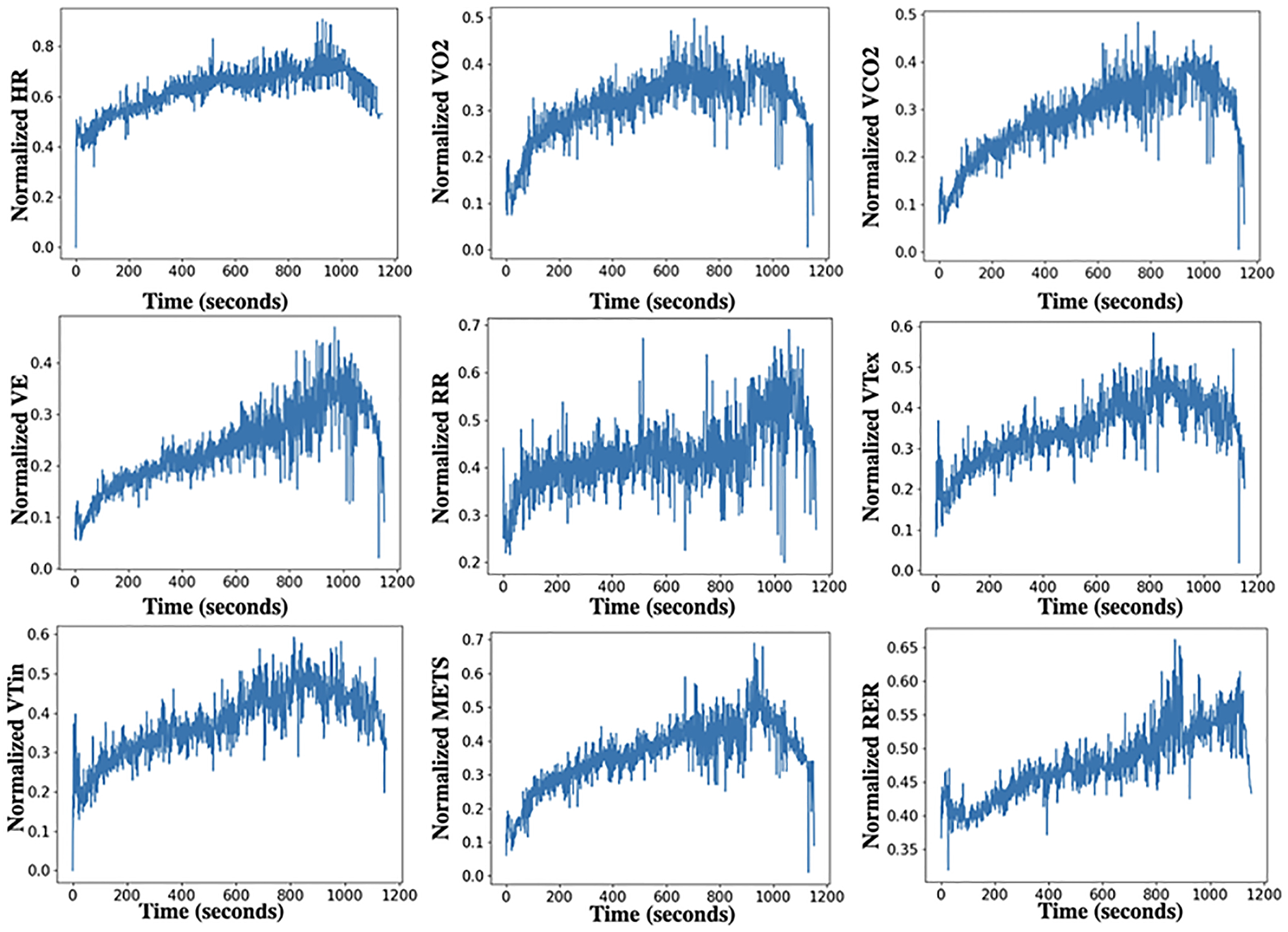
The aggregated second-by-second VE, RER, VTex, VTin, METS, RR, VCO2, VO2, for patients with label HF

**Fig. 4:**
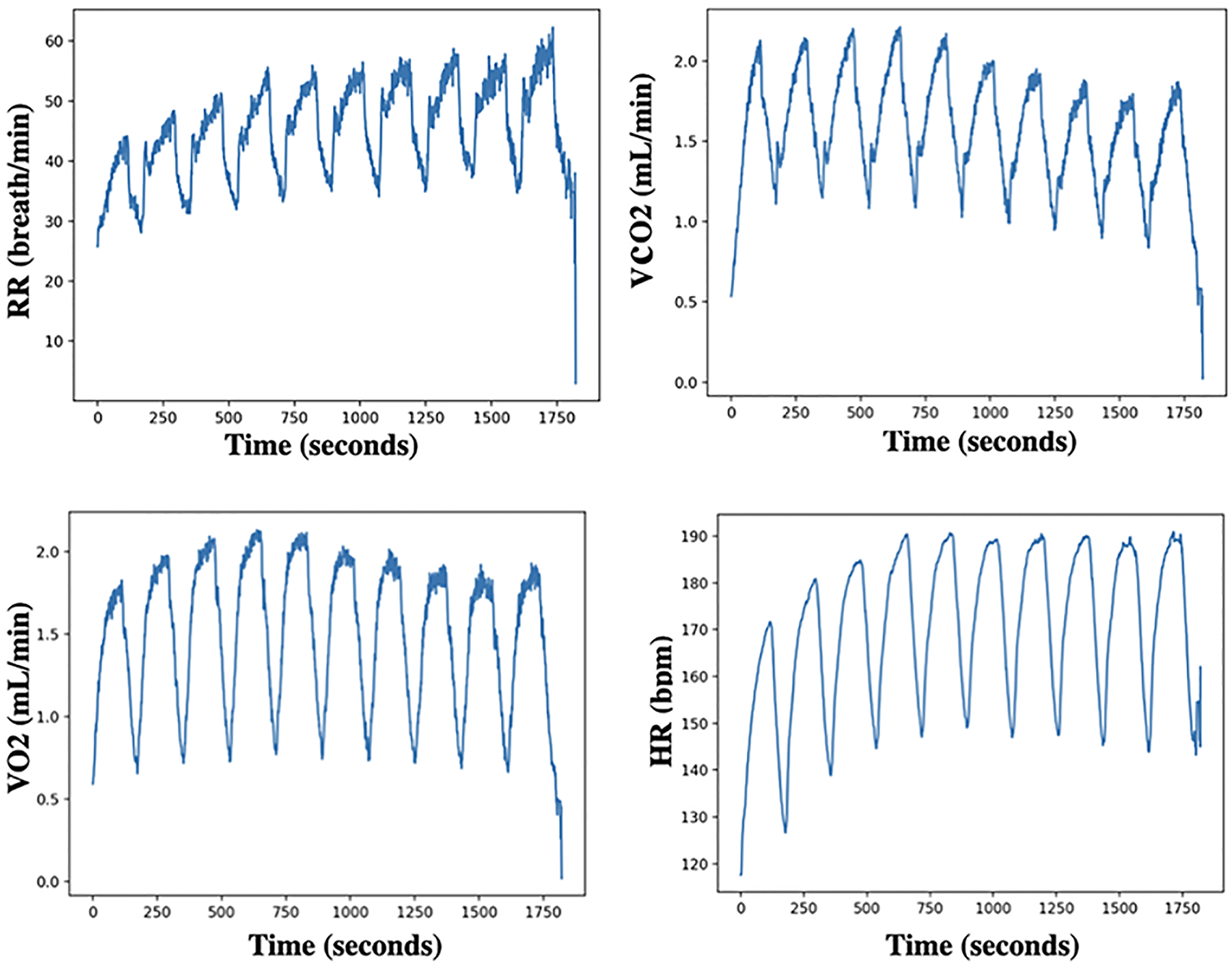
The aggregated second-by-second RR, VCO2, VO2, and HR over all participants from CPET dataset 2

1. *UEA multivariate time series archive*^1^ (Bagnall et al. 2018): The archive includes data sets collected from different applications such as human activity recognition, motion classification, and ECG/EEG signal classification. For variable-length datasets, we pad all series to the same length, setting NaNs for missing observations. When an observation is missing (NaN), the corresponding mask position is set to zero. Also, we noticed inconsistencies between the current ERing dataset available at the UEA multivariate time series archive and the dataset used in the referenced papers Fauvel et al. (2021), Zhang et al. (2020). To ensure the integrity of our experiments, we removed the ERing dataset from our analysis.
2. *Cardiopulmonary exercise testing (CPET) dataset 1* (Brown et al. 2022): The CPET dataset consists of the breath-by-breath readings of 30 patients with two clinically diagnosed conditions: heart failure (HF) and metabolic syndrome (MS) (15 patients each). The testing protocol for gathering data involved using a treadmill with three stages: rest, testing, and recovery. This dataset contains the following variables: metabolic equivalent of task (METS)(1 MET = 3.5 ml/kg/min); heart rate (HR); inspired volumes of oxygen (VO2); expired volumes of carbon dioxide (VCO2); ventilation (VE); respiratory rate (RR); expiratory tidal volume (VTex); and inspiratory tidal volume (VTin); respiratory exchange ratio (RER); speed of the treadmill; elevation of the treadmill; binary outcome variable indicating the clinically diagnosed condition of the patient. The aggregated second-by-second values of normalized CPET variables (i.e. HR, RR, VO2, VE, VCO2, RER, VTin, VTex) for participants with label HF as an example is shown in Fig. 3. In other words, we compute the mean of each CPET variable per second over all participants with the label HF.
3. *Cardiopulmonary exercise testing (CPET) dataset 2* (Coronato et al. 2022): This dataset comprises breath-by-breath readings from 78 healthy children and adolescents who underwent the (multiple brief exercise bouts) (MBEB) task at low, moderate, and high-intensity work rates. Even though all participants completed the ten bouts at low and moderate-tensity, half of them failed and stopped before all ten bouts had been completed (task failure) high-tensity work rate. This dataset the following variables: heart rate (HR); inspired volumes of oxygen (VO2); expired volumes of carbon dioxide (VCO2); respiratory rate (RR); gender; maturational status; body mass; total fat; binary outcome variable indicating whether the participant completed the test. The aggregated second-by-second values of CPET variables (i.e. HR, RR, VO2, VCO2) over all participants are shown in Fig. 4.

### 4.2 Metric

Each model is evaluated using the accuracy score (i.e. $\frac{TP+TN}{TP+FP+TN+FN}$). where TP, FP, TN, and FN are true positive, false positive, true negative, and false negative, respectively.

### 4.3 Friedman test and Wilcoxon test

To find the differences between the methods, we leverage the Freidman test which is a non-parametric statistical test. Moreover, the Wilcoxon-signed rank test is used to compare pairs of classifiers over the datasets. The Friedman test and Wilcoxon-signed rank test with Holm’s α(5%) are conducted by following the process described in (Demšar 2006).

### 4.4 Interpretability

Gradient-weighted class activation mapping (Grad-CAM) (Selvaraju et al. 2017) is one of the well-known methods for generating saliency maps to support convolutional neural network predictions. The Grad-CAM aims to identify the regions of the input data that the most influence the predictions using the class-specific gradient information. In this study, we use the Grad-CAM approach to identify those time steps of the time series that influence the most on the model’s decision for a specifically assigned label. The following paragraph explains how we adapt Grad-CAM for the SupCon-TSC model.

In order to build the attribution map, we apply grad-CAM to the output features of the last 1D convolution layer. First, we compute the importance of each feature map $\left(w_{k}^{c}\right)$ by obtaining the gradient of the output score for specific class $\text{c}\;\left(y_{c}\right)$ with respect to each feature map activation $A^{k}$ as:

(6) $$w_{k}^{c}=\frac{1}{Z}\sum_{i}\frac{\sigma y_{c}}{\sigma A_{i}^{k}}$$

where Z is the total number of units in A. Then, $w_{k}^{c}$ is used to compute a weight combination of feature maps for class c by Eq. (7). The ReLU non-linearity is used to keep only positive values.


(7) $$L_{1D}^{c}=\text{ReLU}\left(\sum_{k}w_{k}^{c}A^{k}\right)$$

### 4.5 Architecture details

The model architecture is as follows:

1. Encoder: ResNet (Wang et al. 2017)
2. Head: two linear layers with ReLu activation function.
3. Classifier: two linear layers with ReLu activation function and Softmax on top.

### 4.6 Hyperparameters

The grid search along with the 5-fold cross-validation on the training set is used to set hyperparameters for each dataset. Please refer to Appendix 1 for the hyperparameters used in our experiments.

### 4.7 Models

We have compared the performance of the proposed method with the following state-of-the-art MTSC models on the UEA Multivariate time series archive datasets.

- *TapNet:* Multivariate time series classification with attentional prototypical network was applied to time series data (Zhang et al. 2020).
- *WEASEL+MUSE (WM):* Word ExtrAction for time Series cLassification plus Multivariate Unsupervised Symbols and dErivatives was applied to time series data (Schäfer and Leser 2017).
- *MLSTM-FCN (MF):* Multivariate LSTM fully convolutional networks for time series classification was applied to time series data (Karim et al. 2019).
- *MTEX-CNN (MC):* Multivariate time series explanations for predictions with convolutional neural networks was applied to time series data (Assaf et al. 2019).
- *CMFM+RF (CMRF):* Random forest (RF) was applied to the set of time series features obtained by complexity measures and features for multivariate time series (CMFMTS) approach (Baldán and Benítez 2021).
- *CMFM+SVM (CMSVM):* Support vector machine (SVM) was applied to the set of time series features obtained by CMFMTS approach (Baldán and Benítez 2021).
- *CMFM+ C5.0B (CMC5.0B):* C5.0 with boosting (C5.0B) was applied to the set of time series features obtained by CMFMTS approach (Baldán and Benítez 2021).
- *CMFM+1NN (CM1NN):* 1-nearest neighbor classifier with Euclidean distance (1NN-ED) was applied to the set of time series features obtained by CMFMTS approach (Baldán and Benítez 2021).
- *XCM:* The eXplainable convolutional neural network model was applied to time series data (Fauvel et al. 2021).
- *LCEM:* Local cascade ensemble for multivariate data classification (LCEM) was applied to time series data (Fauvel et al. 2020).
- *XGBM:* The extreme gradient boosting algorithm was applied to the LCEM transformation (Fauvel et al. 2020).
- *RFM:* Random forest for multivariate (RFM) algorithm was applied to the LCEM transformation (Fauvel et al. 2020).
- *DW*_*I*_ / *DW*_*I*_(*n*) : a 1-nearest neighbor classifier was applied to the sum of DTW distances for each dimension with and without normalization (n) (Shokoohi-Yekta et al. 2017).
- *DW*_*D*_ / *DW*_*D*_(*n*) : Dimension-dependent dynamic time warping (Shokoohi-Yekta et al. 2017) was employed with and without normalization (n). Distances are computed using multidimensional points, and subsequently, a 1-nearest neighbor classifier was applied to them.

### 4.8 Classification performance evaluation

We evaluate the performance of the SupCon-TSC model on two small CPET datasets and the UEA Multivariate time series archive.

#### 4.8.1 CPET datasets

**Fig. 5:**
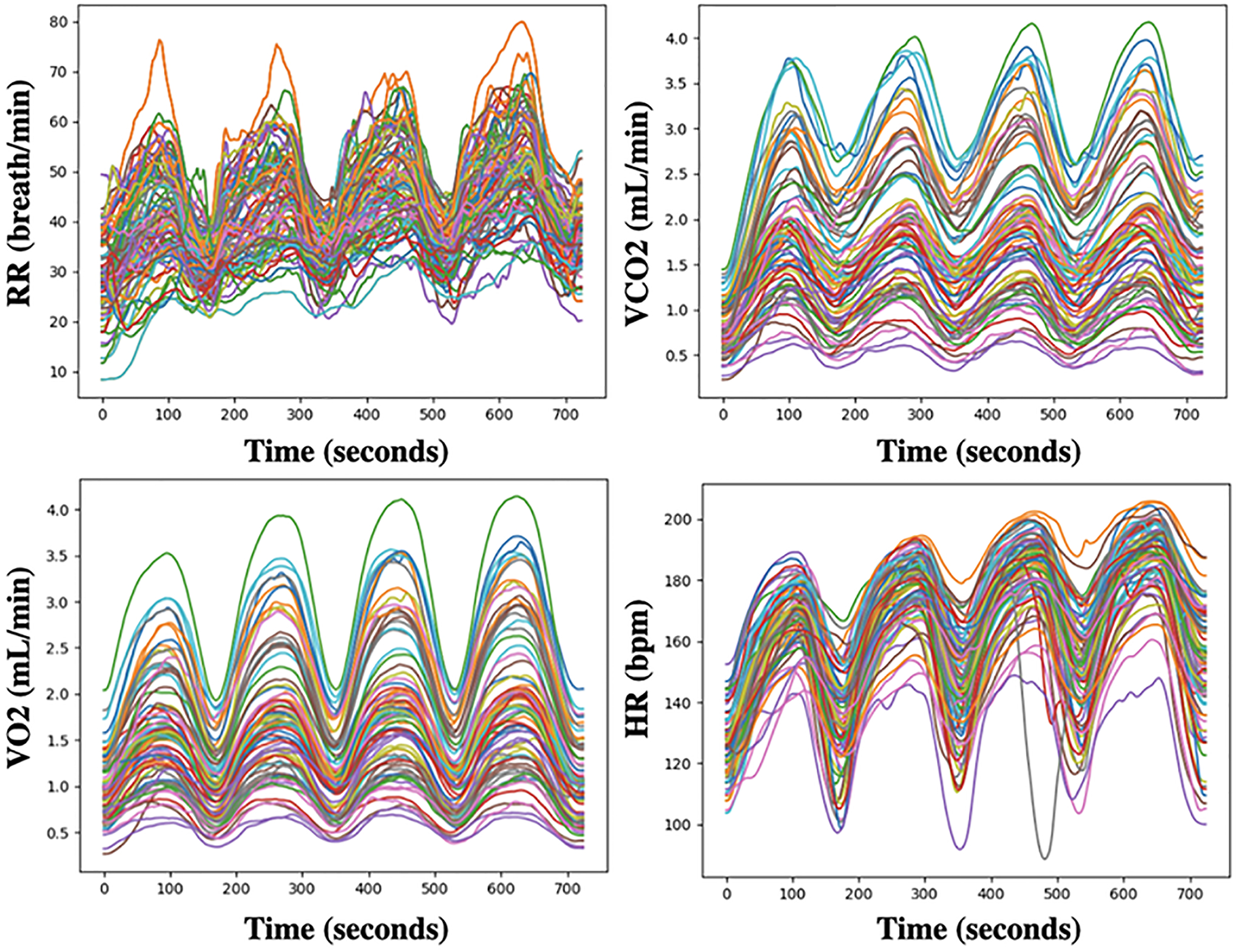
Four bouts of CPET variables after smoothing and aligning the curves

Table 1 shows the performance of the SupCon-TSC alongside the state-of-the-art deep learning models on small CPET datasets 1 and 2. To maintain consistency with prior research (Brown et al. 2022; Coronato et al. 2022), we conducted experimentation through the same k-fold cross-validation method. Additionally, for our experiment, we focused exclusively on the initial four bouts from the second dataset. We then proceeded to smooth and align these bouts as recommended in (Coronato et al. 2022). Four bouts of CPET variables after converting the discrete time series to 78 smoothed and aligned curves are shown in Fig. 5. As shown, the SupCon-TSC model has achieved better accuracy on both datasets. The best accuracy for each dataset is denoted in boldface.

**Fig. 6:**
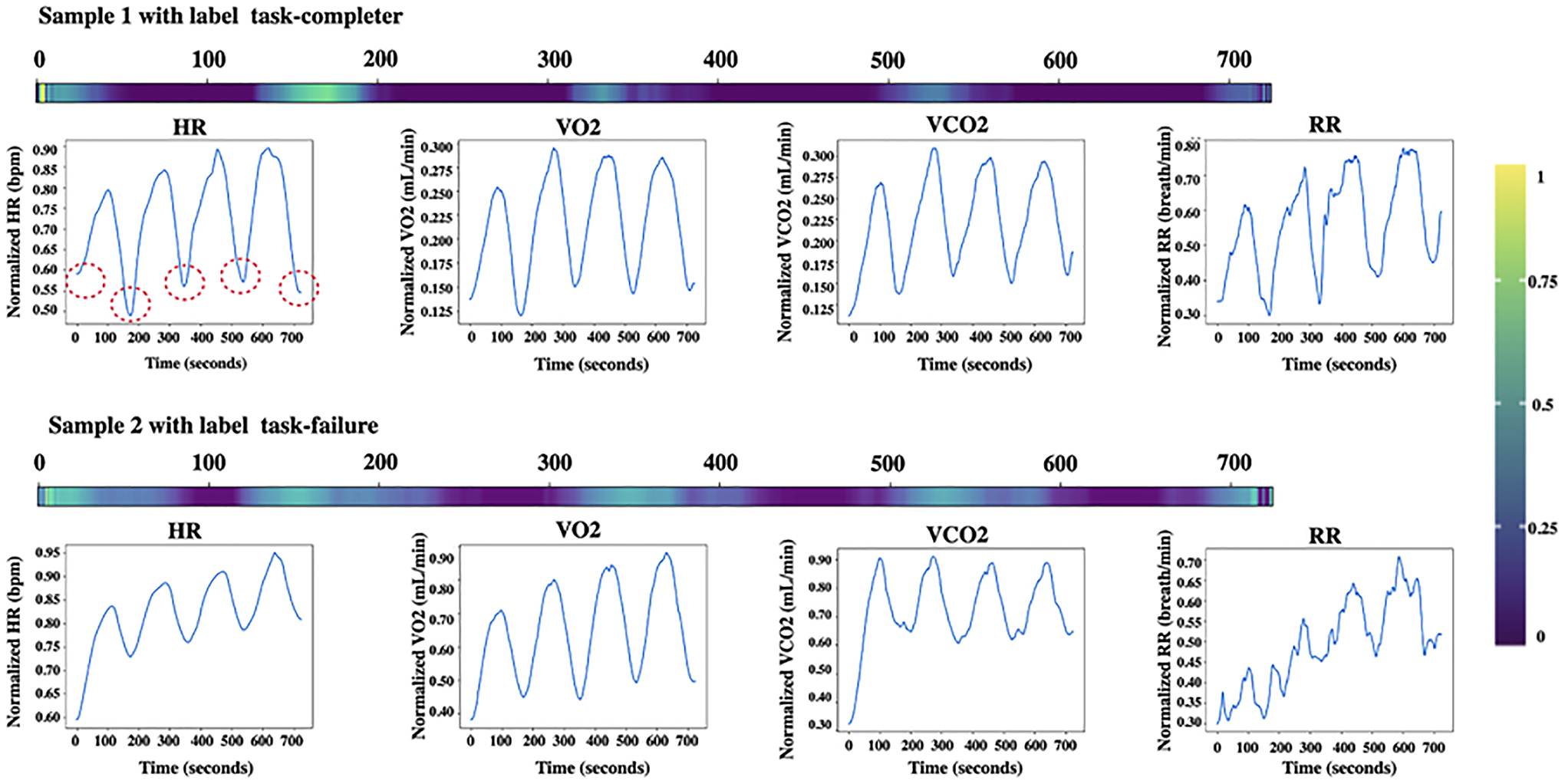
Time attention corresponding to a prediction for two participants with label task-failure and task completer

To investigate the interpretability of the model, we present a comprehensive analysis of the attention mechanism of our SupCon-TSC model when applied to CPET dataset 2. The dataset consists of samples with binary labels indicating whether the participant completed the test. We sought to understand how the model’s attention is distributed across the input data during the prediction process. Figure 6 shows the network’s attention for two samples with different labels from CPET dataset 2. The attention maps provide valuable insights into the regions of interest that the model deems crucial for making predictions. As shown, the network’s attention is spread approximately across time steps 150–190, 310–380, 510–540, and 690–710, which are associated with the valleys in the graphs (i.e., displayed by red circles on the first HR graph). Remarkably, these identified intervals align remarkably well with the recovery points observed in the heart rate (HR) and gas exchange change graphs. From a physiological standpoint, these recovery points have significant implications as they are widely recognized indicators of an individual’s fitness level (Fan et al. (2020); Matsuo et al. (2020)). Notably, we found that the identified recovery points align with the findings from studies Coronato et al. (2022) and Bar-Yoseph et al. (2022). These studies suggest that incomplete recovery from individual exercise bouts may result in a cumulative response deficiency. This deficiency, over time, could potentially manifest in physiological signals that can impact cognitive exercise behavior, which aligns with the patterns identified by the SupCon-TSC model.

#### 4.8.2 UEA multivariate time series archive

The accuracy results of SupCon-TSC and the other state-of-the-art algorithms on the public UEA test sets are presented in Table 2. In the SupCon-TSC approach, ensemble learning is used to make the final prediction by taking the average over the five different models’ outputs trained using 5-fold cross-validation. We perform the hyper-parameter tunning for XCM, TapNet, MTEX-CNN, and MLSTM-FCN models. The results of other baseline models are taken from the Fauvel et al. (2021), and Baldán and Benítez (2021). The dash shows that the approach ran out of memory. Also, the best accuracy for each dataset is boldfaced. The SupCon-TSC was implemented in Python3 using Pytorch 1.10 and all the experiments are conducted on a single Tesla k80 GPU with 11GB memory. As Table 2 indicates, SupCon-TSC achieves better performance on 11 out of 29 UEA datasets in comparison with the baseline methods followed by LCEM with 7 datasets. The average rank is computed using a pairwise Wilcoxon signed rank test and we observe that the best average rank belongs to SupCon-TSC (5.07) which is followed by LCEM (5.26). Furthermore, Table 2 indicates that the SupCon-TSC approach outperforms LCEM methods in 18 out of 29 datasets.

**Fig. 7:**
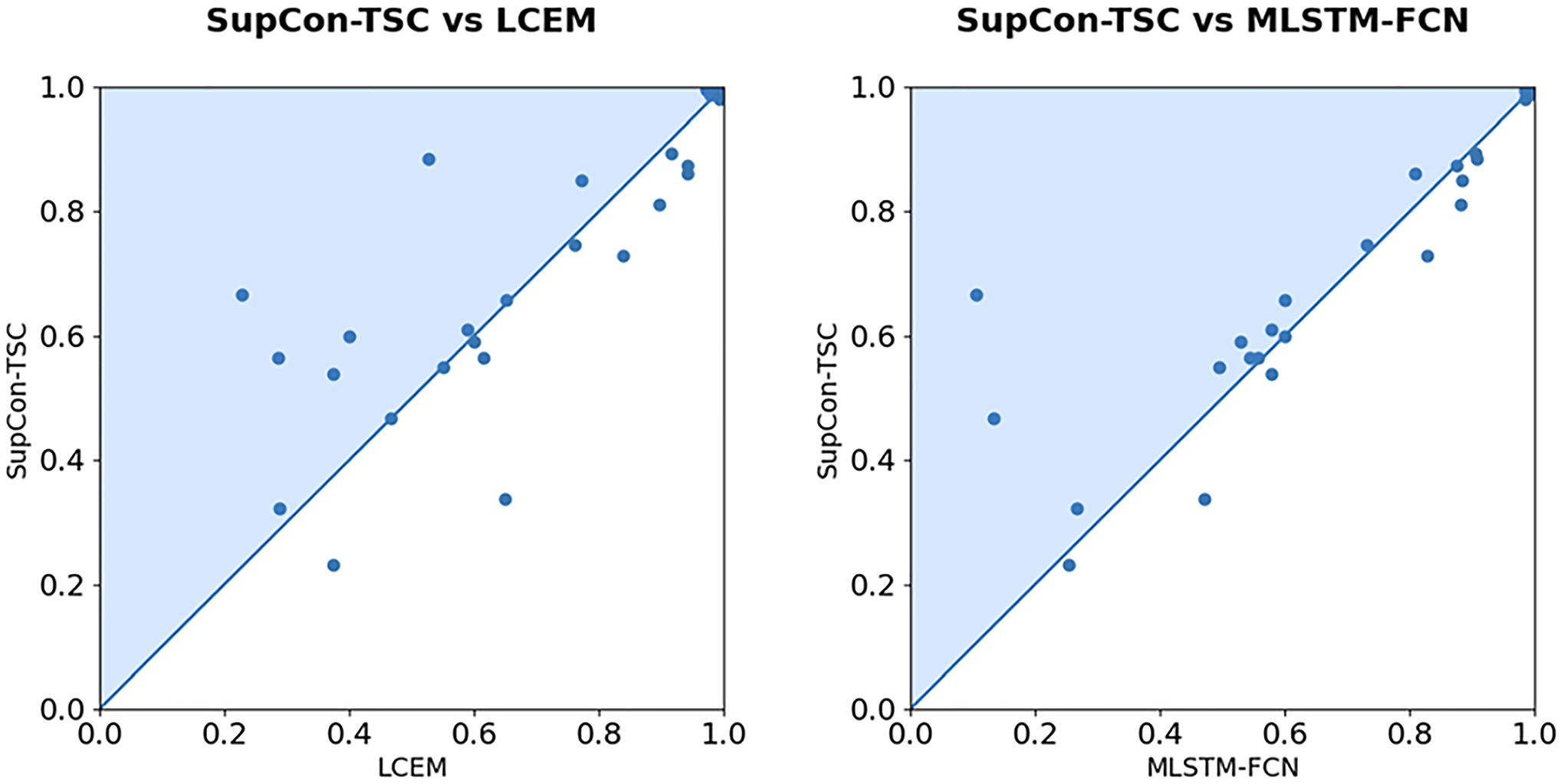
Scatter plots of accuracy on 29 UEA MTSC problems. *Left*: SupCon-TSC vs LCEM showing that SupCon-TSC beats LCEM on 18 problems. *Right*: SupCon-TSC vs MLSTM-FCN showing that SupCon-TSC beats MLSTM-FCN on 19 problems

We applied the Friedman test to investigate if there is a significant difference between the methods. The output of the Friedman test is p=4.205e-19, which is smaller than α=0.05, indicating that there is a significant difference among all ten methods. Figure 7 shows the accuracy scatter plots of SupCon-TSC against each of the LCEM and MLSTM-FCN.

**Fig. 8:**
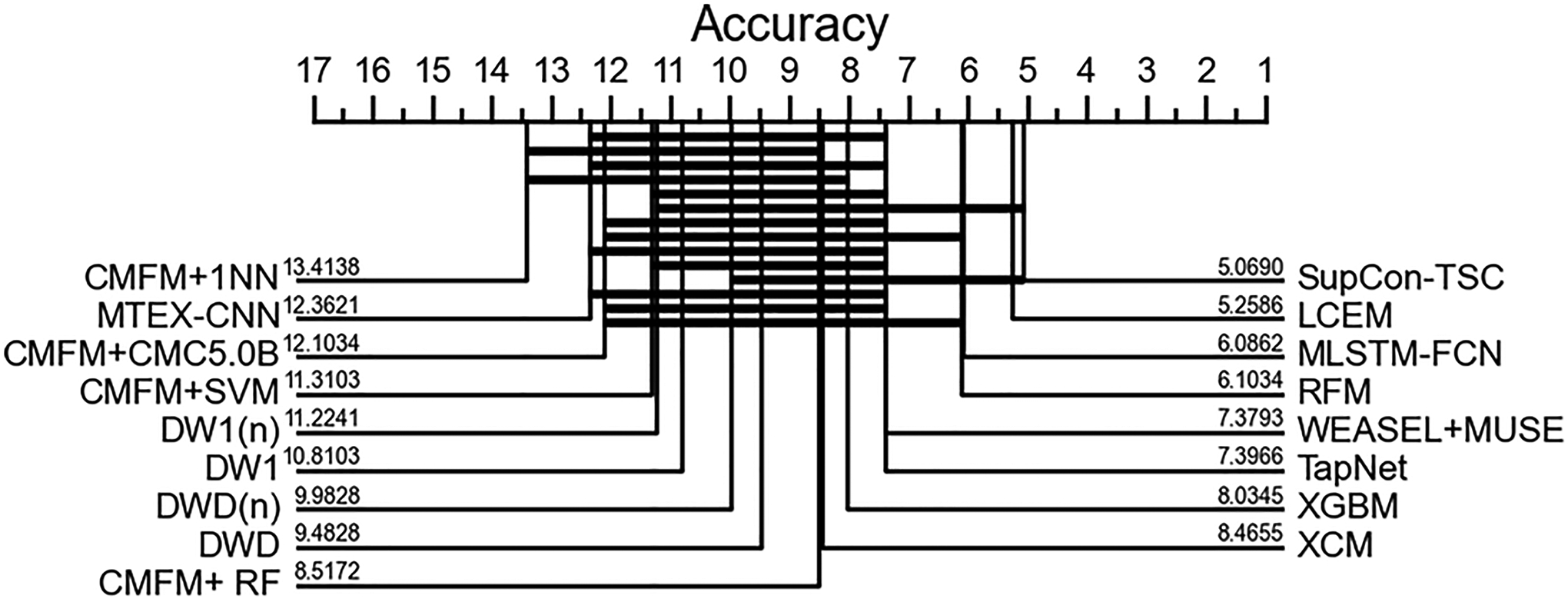
Critical difference diagram (α=0.05)

Figure 8 shows a critical difference diagram obtained by using the pairwise Wilcoxon signed-rank test. The numbers on each line are the average rank of the corresponding method and the solid bars indicate the groups of methods between which there are no significant differences in terms of accuracy. As shown in Fig. 8, the SupCon-TSC model has the first rank followed by LCEM and MLSTM-FCN approaches.

### 4.9 Ablation studies

To study the effect of proposed supervised contrastive learning, we separately train ResNet models with and without proposed supervised contrastive learning. As shown in Table 3, the Supervised Contrastive Learning component improves the performance of the model in 22 out of 29 datasets which verifies the effectiveness of the proposed approach. The best accuracy for each dataset is denoted in boldface.

## 5 Conclusion

This paper has proposed supervised contrastive learning for time series classification (SupCon-TSC). This model is based on the instance-level and cluster-level supervised contrastive learning approaches to learn the discriminative and universal representation for the multivariate time series dataset. As this approach is an end-to-end model, it allows us to detect those time steps of the time series that have the maximum influence on the model’s prediction via utilizing the Grad-CAM method. The experimental results on small CPET datasets indicate the capability of our SupCon-TSC model to learn discriminative features where the labeled dataset is insufficient. Furthermore, the new model outperforms the state-of-the-art models in 11 out of 29 UEA archive datasets. In our future work, we would like to focus on the augmentation methods and evaluate their impact on SupCon-TSC performance.

## Figures and Tables

**Table 1:** The model’s performance on the second CPET datasets 1 and 2

Dataset	Model	k-fold CV	Accuracy (%)
CPET 1	CNN (Brown et al. 2022)	5-fold	90
	SupCon-TSC	5-fold	**97**
CPET 2	GADF + Attention (Coronato et al. 2022)	10-fold	80.8
	SupCon-TSC	10-fold	**86.07**

**Table 2:** Accuracy results on the UEA Multivariate time series datasets. Abbreviations:ST-SupCon-TSC,WM-WEASEL+MUSE, MF-MLSTM-FCN, MC-MTEX-CNN, CMRF-CMFM+RF, CMSVM-CMFM+SVM, CM1NN-CMFM+1NN

Datasets	ST	TapNet	MC	XCM	MF	WM	LCEM	XGBM	RFM
ArticularyWordRecognition (AW)	0.98	0.964	0.913	0.977	0.986	**0.993**	**0.993**	0.99	0.99
AtrialFibrillation (AF)	**0.467**	0.333	0.333	**0.467**	0.133	0.267	**0.467**	0.40	0.333
BasicMotions (BM)	**1**	**1**	0.68	**1**	**1**	**1**	**1**	**1**	**1**
CharacterTrajectories (CT)	**0.997**	**0.997**	0.974	0.995	0.993	0.990	0.979	0.983	0.985
Cricket (C)	**1**	0.958	0.78	0.986	0.986	0.986	0.986	0.972	0.986
DuckDuckGeese (DDG)	0.54	0.44	0.4	0.3	0.579	0.575	0.375	0.40	0.40
EigenWorms (EW)	0.885	0.86	0.419	0.526	**0.908**	0.89	0.527	0.55	**1**
Epilepsy (EP)	0.993	0.978	0.94	0.94	0.985	0.993	0.986	0.978	0.986
EthanolConcentration (EC)	0.231	0.231	0.251	0.32	0.254	0.316	0.372	0.422	**0.433**
FaceDetection (FD)	0.565	0.55	0.50	0.58	0.556	0.545	0.614	**0.629**	0.614
HandMovementDirection (HMD)	0.338	0.37	0.432	0.405	0.472	0.378	**0.649**	0.541	0.50
FingerMovements (FM)	**0.61**	0.52	**0.61**	0.59	0.579	0.54	0.59	0.53	0.56
Handwriting (HW)	0.566	0.37	0.17	0.4	0.544	0.531	0.287	0.267	0.267
Heartbeat (HB)	0.746	0.752	0.721	0.72	0.731	0.727	0.761	0.693	**0.80**
InsectWingbeat (IW)	0.667	0.208	0.105	0.105	0.105	-	0.228	0.237	0.224
JapaneseVowels (JV)	0.987	0.965	0.951	0.986	**0.992**	0.978	0.978	0.968	0.970
Libras (LIB)	0.85	0.877	0.6	0.77	0.883	**0.894**	0.772	0.767	0.783
LSST (LSST)	**0.657**	0.55	0.57	0.51	0.601	0.628	0.652	0.633	0.612
MotorImagery (MI)	0.59	0.53	0.5	0.5	0.529	0.50	**0.60**	0.46	0.55
NATOPS (NATO)	0.894	**0.93**	0.75	0.71	0.905	0.883	0.916	0.90	0.911
PenDigits (PD)	**0.993**	0.98	0.896	0.98	0.99	0.969	0.977	0.951	0.951
PEMS-SF (PEMS)	0.861	0.77	0.838	0.83	0.809	-	0.942	0.983	0.983
PhonemeSpectra (PS)	**0.322**	0.19	0.08	0.13	0.266	0.19	0.288	0.187	0.222
RacketSportsc(RS)	0.875	0.83	0.723	0.78	0.875	0.914	**0.941**	0.928	0.921
SelfRegulationSCP1 (SRS1)	0.73	0.75	0.767	**0.860**	0.829	0.744	0.839	0.829	0.826
SelfRegulationSCP2 (SRS2)	**0.55**	**0.55**	0.50	**0.55**	0.494	0.522	**0.55**	0.483	0.478
SpokenArabicDigits (SA)	**0.995**	0.983	0.986	**0.995**	0.994	0.982	0.973	0.970	0.968
StandWalkJump (SWJ)	**0.6**	0.47	0.4	0.533	**0.6**	0.333	0.40	0.333	0.467
UWaveGestureLibrary (UW)	0.812	0.89	0.69	0.88	0.881	0.903	0.897	0.894	**0.907**
Total best acc	11	4	1	5	4	3	7	2	5
Ours 1-to-1-Wins/ties	-	23	26	24	19	19	18	20	18
Avg. Rank	5.07	7.4	12.36	8.47	6.09	7.37	5.26	8.03	6.11

Datasets	CMRF	CMSVM	CM1NN	CMC5.0B	$DW_{I}$	$DW_{D}$	$DW_{I}(\text{n})$	$DW_{D}(\text{n})$
ArticularyWordRecognition (AW)	0.99	0.977	0.983	0.91	0.98	0.987	0.98	0.987
AtrialFibrillation (AF)	0.20	0.267	0.133	0.20	0.267	0.20	0.267	0.220
BasicMotions (BM)	0.975	0.925	0.95	0.85	**1**	0.975	**1**	0.975
CharacterTrajectories (CT)	0.970	0.970	0.933	0.942	0.969	0.990	0.969	0.989
Cricket (C)	0.972	0.958	0.972	0.861	0.986	**1**	0.986	**1**
DuckDuckGeese (DDG)	0.52	0.44	0.40	0.42	0.55	**0.60**	0.55	**0.60**
EigenWorms (EW)	0.817	0.84	0.794	0.817	0.603	0.618	-	0.618
Epilepsy (EP)	**1**	0.978	0.957	0.884	0.978	0.964	0.978	0.964
EthanolConcentration (EC)	0.335	0.327	0.304	0.35	0.304	0.323	0.304	0.323
FaceDetection (FD)	0.548	0.548	0.579	0.54	0.513	0.529	-	0.529
HandMovementDirection (HMD)	0.284	0.324	0.189	0.338	0.306	0.231	0.306	0.231
FingerMovements (FM)	0.52	0.46	0.53	0.44	0.52	0.53	0.52	0.53
Handwriting (HW)	0.282	0.184	0.249	0.165	0.509	**0.607**	0.316	0.286
Heartbeat (HB)	0.766	0.732	0.62	0.741	0.659	0.717	0.658	0.717
InsectWingbeat (IW)	**0.677**	0.10	0.266	-	-	0.115	-	-
JapaneseVowels (JV)	0.837	0.778	0.695	0.795	0.959	0.949	0.959	0.949
Libras (LIB)	0.867	0.833	0.828	0.839	0.894	0.872	0.894	0.870
LSST (LSST)	0.652	0.648	0.50	0.631	0.575	0.551	0.575	0.551
MotorImagery (MI)	0.51	0.50	0.44	0.49	0.39	0.50	-	0.50
NATOPS (NATO)	0.817	0.75	0.739	0.817	0.85	0.88	0.85	0.883
PenDigits (PD)	0.951	0.959	0.944	0.933	0.939	0.977	0.939	0.977
PEMS-SF (PEMS)	**1**	0.694	0.775	0.965	0.734	0.711	0.734	0.711
PhonemeSpectra (PS)	0.287	0.25	0.158	0.224	0.151	0.151	0.151	0.151
RacketSportsc(RS)	0.809	0.809	0.711	0.728	0.842	0.803	0.842	0.803
SelfRegulationSCP1 (SRS1)	0.812	0.792	0.703	0.812	0.765	0.775	0.765	0.775
SelfRegulationSCP2 (SRS2)	0.417	0.461	0.50	0.539	0.533	0.539	0.533	0.539
SpokenArabicDigits (SA)	0.976	0.979	0.915	0.933	0.960	0.963	0.959	0.963
StandWalkJump (SWJ)	0.333	0.20	0.133	0.257	0.333	0.20	0.333	0.20
UWaveGestureLibrary (UW)	0.772	0.738	0.753	0.641	0.869	0.903	0.868	0.903
Total best acc	3	0	0	0	1	3	1	2
Ours 1-to-1-Wins/ties	21	27	26	26	24	22	21	23
Avg. Rank	8.52	11.31	13.41	12.10	10.81	9.48	11.23	9.98

**Table 3:** Effect of the proposed supervised contrastive learning

Datasets	AW	AF	BM	CT	C	DDG
w/o SupCon	0.97	0.266	**1.0**	0.995	0.986	0.44
w/SupCon	**0.98**	**0.467**	**1.0**	**0.997**	**1.0**	**0.54**
Datasets	EW	EP	EC	FD	HMD	FM
w/o SupCon	0.862	0.985	**0.277**	0.559	**0.378**	0.52
w/ SupCon	**0.885**	**0.993**	0.231	**0.565**	0.338	**0.61**
Datasets	LIB	LSST	MI	NATO	PD	PEMS
w/o SupCon	**0.872**	**0.662**	0.59	**0.911**	0.986	0.843
w/ SupCon	0.85	0.657	**0.59**	0.894	**0.993**	**0.861**
Datasets	HW	HB	IW	JV	PS	SA
w/o SupCon	**624**	0.741	0.665	0.983	0.313	0.993
w/ SupCon	0.566	**0.746**	**0.667**	**0.987**	**0.322**	**0.995**
Datasets	RS	SRS1	SRS2	SWJ	UW	
w/o SupCon	0.848	0.703	0.488	0.333	**0.837**	
w/ SupCon	**0.875**	**0.730**	**0.55**	**0.6**	0.812	

## Data Availability

Open Source UEA Multivariate time series archive Datasets are available at http://timeseriesclassification.com (Bagnall et al. 2018).

## Footnotes

1 Datasets are available at http://timeseriesclassification.com

## Hyperparameters selection

Table shows the hyperparameters used in our experiments.

See Table 4.

**Table 4:** Selected hyperparameters. Abbreviations: $LR_{1^{-}}$ Learning rate 1, $LR_{2^{-}}$ Learning rate 1, $BS_{1^{-}}$ Batch size 1, $BS_{2^{-}}$ Batch size 2

Datasets	$LR_{1}$	$BS_{1}$	Epoch1	$LR_{2}$	$BS_{2}$	Epoch2
ArticularyWordRecognition	0.001	40	100	0.005	20	100
AtrialFibrillation	0.001	15	100	1e-05	15	100
BasicMotions	0.001	10	100	0.001	5	100
CharacterTrajectories	0.001	50	100	0.001	50	100
Cricket	0.001	10	100	0.001	50	100
DuckDuckGeese	0.001	30	100	0.001	5	150
EigenWorms	0.001	10	100	0.001	10	150
Epilepsy	0.001	10	100	0.001	50	150
EthanolConcentration	0.001	10	100	0.001	20	150
FaceDetection	0.001	50	100	0.001	70	100
HandMovementDirection	0.001	50	100	0.0001	5	100
FingerMovements	0.005	100	100	0.0005	100	150
Handwriting	0.001	30	100	0.001	5	150
Heartbeat	0.001	50	100	0.001	10	100
InsectWingbeat	0.001	1000	100	0.0001	1000	100
JapaneseVowels	0.001	20	100	0.001	5	100
Libras	0.0001	30	100	0.001	5	150
LSST	0.001	20	100	0.001	5	100
MotorImagery	0.001	70	100	0.001	10	100
NATOPS	0.005	25	100	0.005	10	100
PenDigits	0.001	100	100	0.001	50	100
PEMS-SF	0.001	70	100	0.001	5	100
Phoneme	0.001	50	100	0.001	200	100
RacketSports	0.001	30	100	1e-05	5	150
SelfRegulationSCP1	0.001	20	100	1e-05	100	100
SelfRegulationSCP2	0.001	20	100	0.0001	5	100
SpokenArabicDigits	0.001	20	100	0.001	10	100
StandWalkJump	0.001	3	100	0.001	9	100
UWaveGestureLibrary	0.001	15	100	0.001	10	150
